# Prediction method of cutting stability of roadheader based on the Newton–Lagrange mixed discrete method

**DOI:** 10.1038/s41598-022-25409-6

**Published:** 2022-12-01

**Authors:** Yun Zhu, Miao Xie, He Wang

**Affiliations:** 1 Department of Mechanical Engineering, Liaoning Technical University, Fuxin, 123000 Liaoning China; 2 Liaoning Provincial Key Laboratory of Large-Scale Mining Equipment, Fuxin, 123000 Liaoning China

**Keywords:** Engineering, Mechanical engineering

## Abstract

In order to study the influence of the multiple interaction of coal and rock and the velocity effect of the cutting head on the flutter stability of the cantilever roadheader, considering the physical characteristics of coal and rock, the structural parameters of the cutting head and the motion parameters of the cutting head, the functional expression of the cutting depth of the cutting head and the participating cutting teeth is fitted, the cutting state mechanical characteristic equation of the dynamic cutting of the cutting teeth is established, and based on the mapping relationship between the cutting teeth and the cutting head, a cutting dynamic model including multiple interactions between cutting head and coal and rock and the speed effect is constructed. An improved discrete method based on Newton–Lagrange mixed interpolation is proposed, and the influence law of the coupling effect of regeneration effect and velocity effect on the stability of cutting flutter under cutting state is clarified. The improved full discrete method is compared with the full discrete method and the semi-discrete method, and the superiority of the improved full discrete method based on the mixed interpolation method is proved. Based on the improved total discrete method, the influence of different cutting system dynamic parameters on the stability is studied. A cutting head coal rock system is built to simulate the flutter of the cutting system. The results show that the improved fully discrete method can reasonably predict the actual cutting state.

## Introduction

With the development of coal mining technology, the demand for the quality of the forming of the sections of the coal mine roadway is gradually increasing. Cantilever roadheader, as vital equipment for intelligent rapid tunneling, has higher requirements for its operational reliability and stability. The cutting mechanism is the key technology for the design and manufacture of roadheader; it has always been a research hotspot in intelligent cutting^1,2^. Hou et al.^3^ made a theoretical study of the force acting on the cutting head of the roadheader and established the vibration equation of the entire roadheader. Li et al.^4^ studied the dynamic behavior of the cutting arm of the roadheader, established the dynamic model of the whole machine based on the Lagrange equation, proposed the virtual excitation method of the external load of the cutting head, and studied the vibration characteristics of the cutting arm. Wei et al.^5^ studied the nonlinear precision and dynamic characteristics of the swing process of the roadheader, and analyzed the cutting performance of the roadheader under different working conditions. Zhang et al.^6^ conducted in-depth research on the mechanical characteristics of the roadheader power system under impact load. Liu et al.^7^ used robot kinematics to conduct a profound study on the impact of the cutting head movement track on the roadway surface topography aspects, and obtained the generation mechanism of the roadway outer contour. Yuan et al.^8^ and others studied the influence of the cutting angle and rotation angle of the pick on the cutting load, load fluctuation, and specific energy consumption of cutting through the method of simulation experiment. Li et al.^9^ studied the influence of helix pick arrangement on the cutting load of the boom type roadheader cutting head, and designed the helix angle and helix number of the cutting head. Wang et al.^10^ established the average and peak cutting force models of pick teeth and the peak cutting force model considering the wear of pick teeth by using the original data of linear full scale cutting and principal component regression analysis and ridge regression analysis. Xu et al.^11^ deeply studied the conversion relationship between the installation angle of the cutting head and the cutting angle, and studied the relationship between the cutting angle and the cutting resistance through numerical simulation. Liu et al.^12^ studied the relationship between the working angle of the pick and the cutting load, obtained the working angle range of the pick, and optimized the working angle of the pick through numerical simulation. Zhang et al.^13^ simplified the movement of the cutting head in swing cutting, studied the influence of cutting depth and cutting speed on cutting performance through numerical simulation, and obtained the optimal cutting depth range. Zhang et al.^13^ Li deeply analyzed the influence of cutting depth and cutting speed on cutting performance through numerical simulation and experimental verification, and determined that cutting depth is a sensitive factor.

The stable working state of the roadheader is an important factor that affects the efficient and reliable operation of the roadheader, and the stable state of the roadheader also has an essential relationship with the cutting vibration of the roadheader. But in recent years, the research on cutting stability is often concentrated on the field of adaptive control. Li et al.^14,15^ Li combines BP neural network with PID control method, through the feedback adjustment method of cutting load and motor current, he realizes the adaptive control of the yaw speed of the roadheader and improves the stability of the roadheader. Liu et al.^16^ proposed an adaptive variable speed cutting control method based on cutting performance optimization by analyzing the working principle of the roadheader, which realized adaptive variable speed cutting in the tunneling process. However, the above research did not carry out theoretical analysis on chatter phenomenon during cutting. There have been few types of research on cutting stability and cutting chatter of cutting head, and most research has focused on the field of metal cutting.

Mann et al.^17,18^ analyzed the stability of the workpiece machining process based on the TFEA theory and made simultaneous predictions of its dynamic machining error. Insperger^19^ and others predicted the stability of the machining process based on the semi-discrete method; Ding et al.^20^ proposed a full discrete prediction method based on the direct integration framework. Li et al.^21,22^ used the second-order semi-discrete method to simultaneously predict the flutter stability region and dynamic machining error. Li et al.^23^ analyzed the discrete characteristics of the dynamic equation of the Euler formula and studied the processing stability of the workpiece based on complete discrete method. Li^24^ proposed the prediction method of processing stability based on the fourth order Runge–Kutta and compared the new method with the complete discrete method. Niu et al.^25^ adopted the generalized Runge–Kutta method and time-domain simulation technology uncover the dynamic mechanism of distinct chatter behaviors in general milling scenarios, and obtained the distribution rule of chatter patterns in stability lobe diagrams for milling processes with general flute-spacing tools considering runout. Xia et al.^26^ improved the full discrete method by Newton and Lagrangian interpolation methods, and discussed the optimal order of interpolation by comparing and verifying the methods. The scholars also studied the prediction of surface roughness in cutting chatter machining through machine learning algorithm. Cruz et al.^27^ predicted the apparent roughness after cutting through two-step machine learning method, and verified the accuracy of the model through experiments. Deshpande et al.^28^ Li predicted the apparent roughness of cutting through artificial neural network combined with cutting force, sound and vibration, and verified the validity of the model by comparing it with the modified regression model.

This paper establishes the force model of the single pick under the cutting process based on the rock-breaking and cutting mechanism of the pick and then constructs the mechanical characteristic equation of the cutting state of dynamic cutting of a single pick, through the functional expression of cutting depth and number of cutting picks involved. Based on the similarity between coal mining and metal cutting fields, this paper introduces the milling chatter model in metal cutting into the coal mine cutting field to analyze the stability of the cutting head; at the same time, an improved fully discrete method based on the Newton–Lagrange hybrid interpolation is proposed to simplify the calculation of vibration equations.

A cutting head mining test bench was built and a cutting stability experiment was carried out to verify the effectiveness of the proposed method. The results show that the Newtonian polynomial prediction method proposed in this paper has faster convergence speed, higher prediction accuracy, and less computational time. It can effectively improve the cutting reliability of the cutting head, improve the smoothness of the coal rock surface after cutting, and reduce the wear of the pick, it can also provide a design basis for the selection of efficient and weak flutter cutting parameters.

## Construction of a dynamic equation for the cutting head-coal rock system

### Force model of the pick

**Figure 1:**
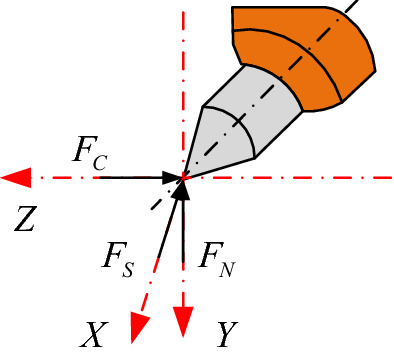
Force on a single pick.

When the pick is wedged into the coal rock, it is subjected to the working resistance opposite to the cutting speed transmitted to it by the coal rock, which is called the cutting resistance F_C._ The cutting teeth press the coal rock and receive the reaction force of the coal rock, which is perpendicular to the direction of the cutting speed and is generated by the feeding action of the cutting part, which is called the feeding resistance F_N_. When the pick cuts into a groove, the coal rock on both sides collapses brittlely. At the same time, the pick receives the reaction force on both sides of the cutting groove, and the force is distributed perpendicular to both sides of the tooth tip, which is called lateral resistance F_S_. The force of the pick in the cutting state is shown in Fig. 1.

The cutting head load can be obtained through the empirical formula^29^. When the roadheader works on the underground coal roadway, the cutting resistance applied to the cutting head can be expressed as:

1 $$ F_{C} = K_{1} + K_{2} h. $$

For the tapered pick, the feeding resistance it receives can be expressed as:

2 $$ F_{N} = K_{3} F_{C} /h^{0.4} . $$

Lateral resistance can be expressed as:

3 $$ F_{S} = F_{C} \left( {\frac{{C_{1} }}{{C_{2} + h}} + C_{3} } \right)\frac{h}{\beta }, $$

where $K_{1} = 0.25P_{k} K_{t} K_{s} K_{a} + 0.1P_{k} S_{T}$, $K_{2} = 0.018P_{k} K_{t} K_{s} K_{a} \beta$, $K_{3} = 2.5 \times \left( {0.15 + 0.00056P_{k} } \right)$; P_k_ = ${44}f^{\frac{3}{2}}$, *f* is the Platts hardness coefficient of rock; *K*_*t*_ = Type factor for picks, 1.5 for tapered teeth, and 1 for cutter teeth; *K*_*s*_ = Pick shape correlation coefficient, $$k_{s} \left\{ {\begin{array}{*{20}l} {k_{ts} k^{\prime}_{ts} k_{td} ,} \hfill & {tapered \, teeth} \hfill \\ {k_{b} k_{a} ,} \hfill & {cutter \, teeth} \hfill \\ \end{array} } \right.$$; $k_{ts}$ = Shape coefficient of the cutter head; $k^{\prime}_{ts}$ = Distribution form coefficient of the cutter bar; $k_{td}$ = Cutter head diameter coefficient; $k_{b}$ = Influence coefficient of the blade width between the blade teeth, which is linearly related to the blade width b*,* it can be expressed as $k_{b} = 0.92 + 0.01b$; $k_{as}$ = Influence coefficient of the shape of the front face of the tooth of the blade, which is 1 for the plane and 0.95 for the oval or pointed shape; *K*_*a*_ = Influence factor of the cutting angle of the pick; $\beta$ = The average stub spacing (mm); *h* = The cutting thickness (mm); *S*_*T*_ = The projected area in the traction direction after the cutting edge is blunt (mm^2^); $m$ = Cutting head number of pick; $n$ = Cutting head speed; $V_{s}$ = Longitudinal feed speed of pick; C_1_, C_2_, C_3_ = cutting pick arrangement factor, where sequential mode $C_{1} = 1.4$,$C_{2} = 0.3$,$C_{3} = 0.15$; Cross mode $C_{1} = 1.0$,$C_{2} = 0.2$,$C_{3} = 0.1$.

The cutting load is mainly affected by the rock property parameters, the structure and shape of the pick, the structure of the cutting head, and the working parameters of the cutting head. However, for the cantilever roadheader, the form of the pick and the overall structure of the cutting head are fixed. At the same time, for coal seams in the same area, the parameters of the coal seam property can be regarded as relatively constant. Therefore, in this case, the cutting load is only related to the cutting thickness h and the operating parameters of the cutting head: *n*, *V*_*s*_*.*

### Force model of cutting head

**Figure 2:**
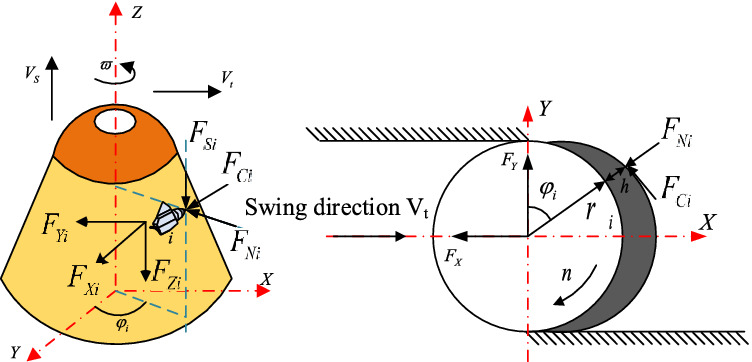
Cutting head load.

Define the rotary center axis of the cutting head as the Z axis and establish the O-XYZ coordinate system as shown in Fig. 2. Due to the complexity of the actual cutting head model, a simplified load diagram of the cutting head is established in Fig. 2. *F*_*Ci*_, *F*_*Ni*_, *F*_*Si*,_ represent the cutting load received by the ith pick of the cutting head. It can be calculated according to formula (1)–(3). $\varpi$ represents the rotation velocity of the cutting head, *V*_*S*_ represents the drilling velocity of the cutting head; V_t_ represents the swing velocity of the cutting head. The triaxial force of the cutting head can be obtained by orthogonal decomposition of cutting resistance, feeding resistance, and lateral resistance.

The three-way component force of the i-th pick on the cutting head can be expressed as:

4 $$ \left\{ {\begin{array}{*{20}l} {F_{Xi} = F_{Ni} \sin \varphi_{i} + F_{Ci} \cos \varphi_{i} } \hfill \\ {F_{Yi} = - F_{Ni} \cos \varphi_{i} + F_{Ci} \sin \varphi } \hfill \\ {F_{Zi} = F_{Si} } \hfill \\ \end{array} } \right.. $$

Since the load of the overall cutting head is closely related to the number of cutting teeth involved in cutting. The number of cutting teeth changes with the drilling depth and the working state of the cutting head.

Assuming that the depth of the cutting head is h_d_ (mm), the number of working teeth that the cutting head participates in is m. Taking the EBZ160 roadheader as an example, the length of the cutting head is 975 mm. According to the design parameters of the pick’s space position, the relationship between the depth of the cutting head and the number of picks is shown in Table 1.

**Table 1:** Relationship between cutting depth and number of cutting teeth.

Number of picks	1	2	3	4	5	6	7	8	9
Cutting depth	0	4	11	20	31	43	58	73	91
Number of picks	10	11	12	13	14	15	16	17	18
Cutting depth	111	132	155	179	205	230	258	287	313
Number of picks	19	20	21	22	23	24	25	26	27
Cutting depth	342	370	399	427	456	484	516	548	580
Number of picks	28	29	30	31	32	33	34	35	36
Cutting depth	610	642	673	705	736	769	808	846	883

The least square method is adapted to fit the drilling depth and the number of working teeth of the cutting head, and the regression equations of the number of cutting teeth in the working area and the drilling depth of the cutting head were obtained under the condition of horizontal swing or vertical swing. Since the number of teeth in the cutting head must be an integer, the regression result needs to be rounded down.

5 $$  m = f(h_{d} )\rfloor= (0.0375h_{d} + 4.96)\rfloor{.} $$

**Figure 3:**
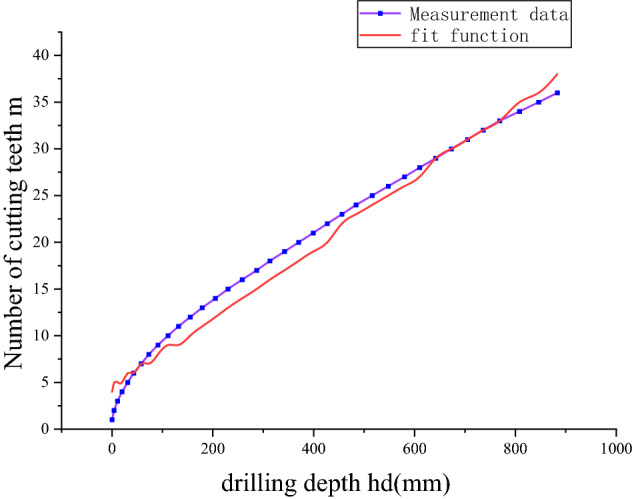
Cutting head load.

The effect of the regression fitting model is shown in Fig. 3. It can be seen from the figure that the fitting function can reflect the expression relationship between the depth of cutting and the number of involved cutting teeth.

To further analyze the fitting effect between the number of cutting head teeth and the depth. We can evaluate the fitting results by goodness of fit (R2) and root mean square error (RMSE), which can be expressed as

6 $$ \begin{array}{*{20}l} {R^{2} = 1 - \frac{{\sum\limits_{i = 1}^{n} {(\hat{y}_{i} - y_{i} )^{2} } }}{{\sum\limits_{i = 1}^{n} {(y_{i} - \overline{y})^{2} } }}} \hfill \\ {RMSE = \sqrt {\frac{1}{n}\sum\limits_{i = 1}^{n} {(\hat{y}_{i} - y_{i} )^{2} } } } \hfill \\ \end{array} , $$

where $y_{i}$, $\hat{y}_{i}$, $\overline{y}$ is the actual value, predicted value and the mean value of actual value, respectively, and n is the total number of the concerned points.

The calculated R2 of the fitted curve is 0.995, which is very close to the constant 1, indicating that the fitted curve has very strong interpretability. The RMSE is 1.55, indicating that the fitting curve has good stability and low dispersion.

The number of cutting teeth on the cutting head is also related to the working state, and it can be expressed by the following formula:

7 $$ N(h_{d} ) = \left\{ {\begin{array}{*{20}l} {f(h_{d} )\rfloor{,}} \hfill & {\text{Cutting head drilling}} \hfill \\ {f(h_{d} )\rfloor/2,} \hfill & {\text{Cutting head swing}} \hfill \\ \end{array} } \right.. $$

**Figure 4:**
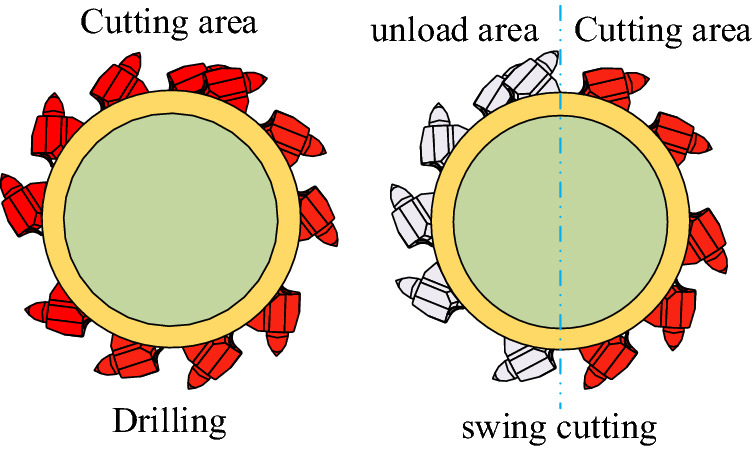
Relationship between cutting depth and number of cutting teeth.

N represents the number of cutting teeth in the cutting area in each working state, which can be shown in Fig. 4.

Based on the theory of force line translation, the three-direction forces in the vertical, horizontal, and axial directions on the cutting head can be obtained by vector summing of the forces on the picks located in the cutting area at the same time:

The cross-cutting resistance of the cutting head along the swing direction can be expressed as:

8 $$ \begin{aligned} F_{X} = & \sum\limits_{i = 1}^{{N(h_{d} )}} {(F_{Ni} \sin \varphi_{i} + F_{Ci} \cos \varphi_{i} )} \\ = & \sum\limits_{i = 1}^{{N(h_{d} )}} {((K_{3} (K_{1} + K_{2} h)/h^{0.4} )\sin \varphi_{i} + (K_{1} + K_{2} h)\cos \varphi_{i} )} . \\ \end{aligned} $$

The vertical load of the cutting head can be expressed as:

9 $$ \begin{aligned} F_{Y} = & \sum\limits_{i = 1}^{{N(h_{d} )}} {( - F_{Ni} \cos \varphi_{i} + F_{Ci} \sin \varphi )} \\ = & \sum\limits_{i = 1}^{{N(h_{d} )}} {( - (K_{3} (K_{1} + K_{2} h)/h^{0.4} )\cos \varphi_{i} + (K_{1} + K_{2} h)\sin \varphi_{i} )} . \\ \end{aligned} $$

The cutting head drilling resistance can be expressed as:

10 $$ F_{Z} = \sum\limits_{i = 1}^{{N(h_{d} )}} {F_{Si} } = \sum\limits_{i = 1}^{{N(h_{d} )}} {(K_{1} + K_{2} h)\left( {\frac{{C_{1} }}{{C_{2} + h}} + C_{3} } \right)\frac{h}{\beta }} . $$

### Dynamic equation of cutting head

**Figure 5:**
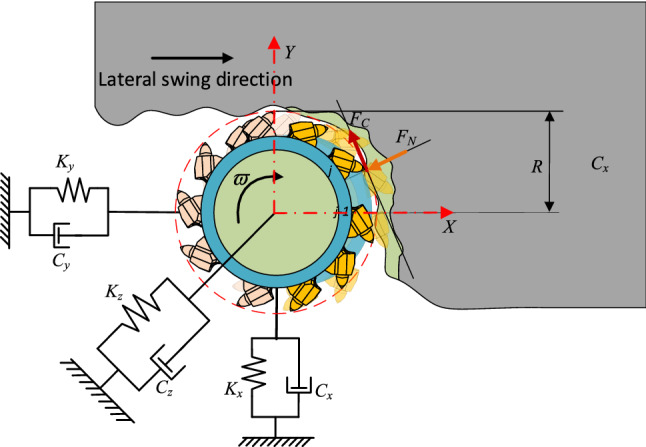
Dynamic model of cutting head system.

Assuming that the vibration amplitude of the cutting head of the longitudinal axis roadheader is within the elastic deformation range, according to the superposition principle, the vibration of the cutting head can be regarded as the superposition of the individual axial forces in the three directions of *F*_*x*_, *F*_*y*_, *F*_*z*_. The dynamic model of the cutting head system during the coal cutting process can be shown in Fig. 5.

The dynamic equation of the cutting motion of the cutting head can be expressed as:

11 $$ M\ddot{s}(t) + C\dot{s}(t) + Ks(t) = F(h,t), $$

where

$$ M = \left[ {\begin{array}{*{20}c} {m_{x} } & {} & {} \\ {} & {m_{y} } & {} \\ {} & {} & {m_{z} } \\ \end{array} } \right],\;K{ = }\left[ {\begin{array}{*{20}c} {m_{x} \omega_{nx}^{2} } & {} & {} \\ {} & {m_{y} \omega_{ny}^{2} } & {} \\ {} & {} & {m_{z} \omega_{nz}^{2} } \\ \end{array} } \right],\;C{ = }\left[ {\begin{array}{*{20}c} {2m_{x} \zeta_{x} \omega_{nx} } & {} & {} \\ {} & {2m_{y} \zeta_{y} \omega_{ny} } & {} \\ {} & {} & {2m_{z} \zeta_{z} \omega_{nz} } \\ \end{array} } \right],\;s(t) = \left[ {\begin{array}{*{20}c} {x(t)} \\ {y(t)} \\ {z(t)} \\ \end{array} } \right], $$

where M, C, K are the modal mass matrix, damping matrix, and stiffness matrix of the cutting head; $m_{x}$, $m_{y}$, $m_{z}$, $\zeta_{x}$, $\zeta_{{\text{y}}}$, $\zeta_{{\text{z}}}$, $\omega_{nx}$, $\omega_{{n{\text{y}}}}$, $\omega_{nz}$ are the modal mass, damping ratio, and natural frequency in the x, y, and z directions; *s(t)* represents the vibration displacement of the tool at the current moment;

$F(h,t)$ is the cutting load experienced by the cutting head. It is related to the cutting thickness and the time consumption. It can be expressed as:

12 $$ F(h,t){ = }\left[ {\begin{array}{*{20}c} {\sum\limits_{i = 1}^{{N(h_{d} )}} {g(\varphi_{i} (t))((K_{3} (K_{1} + K_{2} h)/h^{0.4} )\sin \varphi_{i} (t) + (K_{1} + K_{2} h_{i} (t))\cos \varphi_{i} (t))} } \\ {\sum\limits_{i = 1}^{{N(h_{d} )}} {g(\varphi_{i} (t))( - (K_{3} (K_{1} + K_{2} h)/h^{0.4} )\cos \varphi_{i} (t) + (K_{1} + K_{2} h_{i} (t))\sin \varphi_{i} (t))} } \\ {\sum\limits_{i = 1}^{{N(h_{d} )}} {g(\varphi_{i} (t))(K_{1} + K_{2} h_{i} (t))(\frac{{C_{1} }}{{C_{2} + h_{i} (t)}} + C_{3} )\frac{{h_{i} (t)}}{\beta }} } \\ \end{array} } \right], $$

where $\varphi_{i} (t)$ is the cutting angle at which the pick participates in cutting:

13 $$ \varphi_{i} (t) = (2\pi {\text{n}}/60)t + (i - 1)2\pi /m. $$

$g(\varphi_{i} (t))$ is the piecewise function for judging the cutting process of the pick:

14 $$ g(\varphi_{i} (t)) = \left\{ {\begin{array}{*{20}c} 1 \\ 0 \\ \end{array} } \right.{\kern 1pt} {\kern 1pt} {\kern 1pt} {\kern 1pt} {\kern 1pt} {\kern 1pt} {\kern 1pt} {\kern 1pt} \begin{array}{*{20}l} {\omega_{in} \le \omega_{i} \le \omega_{out} } \hfill \\ {others} \hfill \\ \end{array} , $$

where $\omega_{{{\text{in}}}}$ is the starting cutting position of the i-th pick, $\omega_{{{\text{out}}}}$ is the end cutting position; For counterclockwise cutting $\omega_{{{\text{out}}}} = {\text{arcos(1}} - {\text{a}}_{{\text{e}}} {\text{/R)}}$, For clockwise cutting, $\omega_{{{\text{in}}}} = {\text{arcos(1}} - {\text{a}}_{{\text{e}}} {\text{/R)}}$, $\omega_{{{\text{out}}}} = \pi$, where ${\text{a}}_{{\text{e}}}$ is the radial depth of cut, R is the radius of the cutting head.

**Figure 6:**
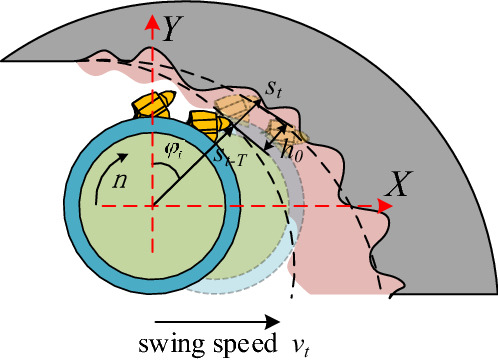
Dynamic cutting thickness change.

*h(t)* Indicates dynamic cutting thickness:

15 $$ h_{i} (t) = h_{0} + [s_{i} (t - T) - s_{i} (t)], $$

where *h*_*0*_ represents the theoretical cutting thickness, *s*_i_(*t*), *s*_*i*_(*t−T*), respectively represent the dynamic displacement of the pick at the current time t and the dynamic displacement of the pick at the corresponding time (t − T) one cycle ahead, as shown in Fig. 6.

Since the change of cutting thickness is only reflected in the x and y directions of the cutting head, therefore, the dynamic cutting thickness $\Delta h = [s_{i} (t - T) - s_{i} (t)]$ can be expressed by the dynamic displacement of the pick:

16 $$ \Delta h{ = } - \Delta x\sin \varphi_{i} (t) - \Delta y\cos \varphi_{i} (t). $$

To simplify the solution of the dynamic equation, take the Taylor expansion for the parametric terms with *h*_*i*_*(t)* in Eq. (11):

17 $$ \begin{aligned} h_{i} (t)^{ - 0.4} = & \left( {h_{0} + \Delta h} \right)^{ - 0.4} = h_{0}^{ - 0.4} \left( {1 + \frac{\Delta h}{{h_{0} }}} \right)^{ - 0.4} \\ = & h_{0}^{ - 0.4} \left( {1 - 0.4\frac{\Delta h}{{h_{0} }} + o(\Delta h^{2} )} \right) \\ \frac{{C_{1} }}{{C_{2} + h_{i} (t)}} = & \frac{{C_{1} }}{{C_{2} + h_{0} + \Delta h}} = \left( {\frac{{C_{1} }}{{C_{2} + h_{0} }}} \right)\frac{1}{{1 + \frac{\Delta h}{{C_{2} + h_{0} }}}} \\ = & \left( {\frac{{C_{1} }}{{C_{2} + h_{0} }}} \right)\left( {1 - \frac{\Delta h}{{C_{2} + h_{0} }} + o(\Delta h^{2} )} \right). \\ \end{aligned} $$

Since the dynamic displacement is relatively small and can be ignored, higher-order terms in the expansion are discarded.

Bring Eqs. (14)–(16) into Eq. (11) the cutting load is approximated by:

18 $$ F(h,t) = \left\{ {\begin{array}{*{20}c} {F_{x} } \\ {F_{y} } \\ {F_{z} } \\ \end{array} } \right\} = \left[ {\begin{array}{*{20}c} {\alpha_{xx} } & {\alpha_{xy} } & 0 \\ {\alpha_{yx} } & {\alpha_{yy} } & 0 \\ {\alpha_{zx} } & {\alpha_{zy} } & 0 \\ \end{array} } \right]\left\{ {\begin{array}{*{20}c} {\Delta x} \\ {\Delta y} \\ {\Delta z} \\ \end{array} } \right\} + \left[ {\begin{array}{*{20}c} {\sum\limits_{i = 1}^{{N(h_{d} )}} {K_{1} \cos \varphi_{i} (t)} } \\ {\sum\limits_{i = 1}^{{N(h_{d} )}} {K_{1} \sin \varphi_{i} (t)} } \\ 0 \\ \end{array} } \right] + \left[ {\begin{array}{*{20}c} {F_{x0} } \\ {F_{y0} } \\ {F_{z0} } \\ \end{array} } \right], $$

where $\alpha_{xx} ,\;\alpha_{xy} ,\;\alpha_{yx} ,\;\alpha_{yy} ,\;\alpha_{zx} ,\;\alpha_{zy}$ represents the time-varying direction coefficient:

19 $$ \left\{ {\begin{array}{*{20}l} {\alpha_{xx} = \sum\limits_{i = 1}^{{N(h_{d} )}} {g(\varphi_{i} (t))( - 0.4K_{1} K_{3} h_{0}^{ - 1.4} \sin \varphi_{i} (t) + K_{2} \cos \varphi_{i} (t))\sin \varphi_{i} (t)} } \hfill \\ {\alpha_{xy} = \sum\limits_{i = 1}^{{N(h_{d} )}} {g(\varphi_{i} (t))( - 0.4K_{1} K_{3} h_{0}^{ - 1.4} \sin \varphi_{i} (t) + K_{2} \cos \varphi_{i} (t))\cos \varphi_{i} (t)} } \hfill \\ {\alpha_{yx} = \sum\limits_{i = 1}^{{N(h_{d} )}} {g(\varphi_{i} (t))(0.4K_{1} K_{3} h_{0}^{ - 1.4} \cos \varphi_{i} (t) + K_{2} \sin \varphi_{i} (t))\sin \varphi_{i} (t)} } \hfill \\ {\alpha_{yy} = \sum\limits_{i = 1}^{{N(h_{d} )}} {g(\varphi_{i} (t))(0.4K_{1} K_{3} h_{0}^{ - 1.4} \cos \varphi_{i} (t) + K_{2} \sin \varphi_{i} (t))\cos \varphi_{i} (t)} } \hfill \\ {\alpha_{zx} = \sum\limits_{i = 1}^{{N(h_{d} )}} {g(\varphi_{i} (t))(\frac{{2C_{1} C_{2} K_{2} h_{0} + C_{1} C_{2} K_{1} + C_{1} K_{2} h_{0}^{2} }}{{\beta (C_{2} + h_{0} )^{2} }})\sin \varphi_{i} (t)} } \hfill \\ {\alpha_{zy} = \sum\limits_{i = 1}^{{N(h_{d} )}} {g(\varphi_{i} (t))(\frac{{2C_{1} C_{2} K_{2} h_{0} + C_{1} C_{2} K_{1} + C_{1} K_{2} h_{0}^{2} }}{{\beta (C_{2} + h_{0} )^{2} }})\cos \varphi_{i} (t)} } \hfill \\ \end{array} .} \right. $$

Suppose $$K_{c} (t) = \left[ {\begin{array}{*{20}c} {\alpha_{xx} } & {\alpha_{xy} } & 0 \\ {\alpha_{yx} } & {\alpha_{yy} } & 0 \\ {\alpha_{zx} } & {\alpha_{zy} } & 0 \\ \end{array} } \right]$$, $$b_{0} (t) = \left[ {\begin{array}{*{20}c} {\sum\limits_{i = 1}^{{N(h_{d} )}} {K_{1} \cos \varphi_{i} (t)} } \\ {\sum\limits_{i = 1}^{{N(h_{d} )}} {K_{1} \sin \varphi_{i} (t)} } \\ 0 \\ \end{array} } \right]$$, $$F_{0} (t) = \left[ {\begin{array}{*{20}c} {F_{x0} } \\ {F_{y0} } \\ {F_{z0} } \\ \end{array} } \right]$$.

The dynamic equation of the cutting head can be expressed as the following delay differential equation:

20 $$ M\ddot{s}(t) + C\dot{s}(t) + Ks(t) = K_{c} (t)(s(t) - s(t - T)) + F_{0} (t) + b_{0} \left( t \right). $$

Since the simplified cutting force model is linear, the static quantity irrelevant to the dynamic displacement can be ignored when calculating the regenerative flutter stability of the cutting head. Simplified system can be obtained:

21 $$ M\text{ }\ddot{s}(t) + C\dot{s}(t) + Ks(t) = K_{c} (t)(s(t) - s(t - T)). $$

## Prediction of cutting stability of the cutting head-coal system

### Cutting stability prediction based on the Newton–Lagrange hybrid interpolation method

A three-DOF milling model can be obtained in state-space from Eq. (20).

22 $$ \dot{\gamma }(t) = A_{0} \gamma (t) + A(t)\gamma (t - T) + B(t)\gamma (t - T), $$

with:

23 $$ \begin{gathered} \eta (t) = M\dot{s}(t) + Cs(t)/2 \hfill \\ \gamma (t) = \left[ {\begin{array}{*{20}c} {s(t)} & {\eta (t)} \\ \end{array} } \right]^{T} , \hfill \\ \end{gathered} $$

24 $$ \begin{gathered} A_{0} = \left[ {\begin{array}{*{20}c} { - M^{ - 1} C/2} & {M^{ - 1} } \\ {CM^{ - 1} C/4 - K} & { - CM^{ - 1} /2} \\ \end{array} } \right] \hfill \\ \begin{array}{*{20}c} {A(t) = \left[ {\begin{array}{*{20}c} 0 & 0 \\ {K_{c} (t)} & 0 \\ \end{array} } \right]} & {B(t) = \left[ {\begin{array}{*{20}c} 0 & 0 \\ { - K_{c} (t)} & 0 \\ \end{array} } \right]} \\ \end{array} . \hfill \\ \end{gathered} $$

The expression of the state space can be solved by the direct integration method, and the time period T is discretized into m parts, then the response of Eq. (21) can be expressed as:

25 $$ \gamma (t) = e^{{A_{0} (t - k\tau )}} \gamma (k\tau ) + \int_{k\tau }^{t} {\left\{ {e^{{A_{0} (t - \delta )}} [A(\delta )\gamma (\delta ) + B(\delta )\gamma (\delta - T)]} \right\}} d\delta . $$

When the time is $t = (k + 1)\tau$, the corresponding response $\gamma_{k + 1}$ is

26 $$ \gamma_{k + 1} = e^{{A_{0} \tau }} \gamma (k\tau ) + \int_{0}^{k\tau } {\left\{ {e^{{A_{0} \delta }} \left[ {\begin{array}{*{20}l} {A(k\tau + \tau - \delta )\gamma (k\tau + \tau - \delta ) + } \hfill \\ {B(k\tau + \tau - \delta )\gamma (k\tau + \tau - \delta - T)} \hfill \\ \end{array} } \right]} \right\}} d\delta . $$

The integral term in Eq. (25) is approximated by Newton interpolation and Lagrangian interpolation respectively, $A(k\tau + \tau - \delta )$ and $B(k\tau + \tau - \delta )$ in Eq. (25) is approximated by the first-order Lagrangian method interpolates based on the interval endpoints $A_{{\text{k + 1}}}$, $A_{{\text{k}}}$.

27 $$ \begin{gathered} A(k\tau + \tau - \delta ) \doteq A_{{\text{k + 1}}} + (A_{{\text{k}}} - A_{{\text{k + 1}}} )\delta /\tau , \hfill \\ B(k\tau + \tau - \delta ) \doteq B_{{\text{k + 1}}} + (B_{{\text{k}}} - B_{{\text{k + 1}}} )\delta /\tau . \hfill \\ \end{gathered} $$

$\gamma (k\tau + \tau - \delta )$ in Eq. (25) is approximated by the third-order Newton interpolation polynomial, four endpoints $\gamma_{k + 1}$, $\gamma_{k}$, $\gamma_{k - 1}$, $\gamma_{k - 2}$ are used to interpolate calculation.

28 $$ \gamma (k\tau + \tau - \xi ) = a_{0} \gamma_{k + 1} + b_{0} \gamma_{k} + c_{0} \gamma_{k - 1} + d_{0} \gamma_{k - 2} , $$

where:

29 $$ \begin{array}{*{20}c} {a_{0} = - \frac{{\delta^{3} }}{{6\tau^{3} }} + \frac{{\delta^{2} }}{{\tau^{2} }} - \frac{11\delta }{{6\tau }} + 1} & {b_{0} = \frac{{\delta^{3} }}{{2\tau^{3} }} - \frac{{5\delta^{2} }}{{2\tau^{2} }} + \frac{3\delta }{\tau }} \\ {c_{0} = - \frac{{\delta^{3} }}{{2\tau^{3} }} + \frac{{2\delta^{2} }}{{\tau^{2} }} - \frac{3\delta }{{2\tau }}} & {d_{0} = \frac{{\delta^{3} }}{{6\tau^{3} }} - \frac{{\delta^{2} }}{{2\tau^{2} }} + \frac{\delta }{3\tau }} \\ \end{array} . $$

The delay term $\gamma (k\tau + \tau - \delta - T)$ is approximated by the Lagrangian polynomial. When using the first-order Lagrangian method, the discrete map can be obtained:

30 $$ \gamma (k\tau + \tau - \xi - T) = \frac{\delta }{2}\gamma_{k - m} + \left( {1 - \frac{\delta }{2}} \right)\gamma_{k - m + 1} . $$

Equation (25) can be obtained using Eqs. (26)–(29):

31 $$ \left( {I - F_{k1} } \right)\gamma_{k + 1} = \left( {F_{0} + F_{k} } \right)\gamma_{k} + F_{kp1} \gamma_{k - 1} + F_{kp2} \gamma_{k - 2} + F_{k1m} \gamma_{k + 1 - m} + F_{km} \gamma_{k - m} . $$

With:

32 $$ \begin{array}{*{20}l} {\mathbf{F}}_{k1} = \left( {{{\varvec{\Psi}}}_{1} - \frac{{11{{\varvec{\Psi}}}_{2} }}{6\tau } + \frac{{{{\varvec{\Psi}}}_{3} }}{{\tau^{2} }} - \frac{{{{\varvec{\Psi}}}_{4} }}{{6\tau^{3} }}} \right)A_{{\text{k + 1}}} + \left( {{{\varvec{\Psi}}}_{2} - \frac{{11{{\varvec{\Psi}}}_{3} }}{6\tau } + \frac{{{{\varvec{\Psi}}}_{4} }}{{\tau^{2} }} - \frac{{{{\varvec{\Psi}}}_{5} }}{{6\tau^{3} }}} \right)(A_{{\text{k}}} - A_{{\text{k + 1}}} )/\tau \hfill \\ {\mathbf{F}}_{k} = \left( {\frac{{3{{\varvec{\Psi}}}_{2} }}{\tau } - \frac{{5{{\varvec{\Psi}}}_{3} }}{{2\tau^{2} }} + \frac{{{{\varvec{\Psi}}}_{4} }}{{2\tau^{3} }}} \right)A_{{\text{k + 1}}} + \left( {\frac{{3\Psi_{3} }}{\tau } - \frac{{5{{\varvec{\Psi}}}_{4} }}{{2\tau^{2} }} + \frac{{{{\varvec{\Psi}}}_{5} }}{{2\tau^{3} }}} \right)(A_{{\text{k}}} - A_{{\text{k + 1}}} )/\tau \hfill \\ {\mathbf{F}}_{kp1} = \left( { - \frac{{3{{\varvec{\Psi}}}_{2} }}{2\tau } + \frac{{2{{\varvec{\Psi}}}_{3} }}{{\tau^{2} }} - \frac{{{{\varvec{\Psi}}}_{4} }}{{2\tau^{3} }}} \right)A_{{\text{k + 1}}} + \left( { - \frac{{3{{\varvec{\Psi}}}_{3} }}{2\tau } + \frac{{2{{\varvec{\Psi}}}_{4} }}{{\tau^{2} }} - \frac{{{{\varvec{\Psi}}}_{5} }}{{2\tau^{3} }}} \right)(A_{{\text{k}}} - A_{{\text{k + 1}}} )/\tau \hfill \\ {\mathbf{F}}_{kp2} = \left( {\frac{{{{\varvec{\Psi}}}_{2} }}{3\tau } - \frac{{{{\varvec{\Psi}}}_{3} }}{{2\tau^{2} }} + \frac{{{{\varvec{\Psi}}}_{4} }}{{6\tau^{3} }}} \right)A_{{\text{k + 1}}} + \left( {\frac{{{{\varvec{\Psi}}}_{3} }}{3\tau } - \frac{{{{\varvec{\Psi}}}_{4} }}{{2\tau^{2} }} + \frac{{{{\varvec{\Psi}}}_{5} }}{{6\tau^{3} }}} \right)(A_{{\text{k}}} - A_{{\text{k + 1}}} )/\tau \hfill \\ {\mathbf{F}}_{k1m} = \left( {{{\varvec{\Psi}}}_{1} - \frac{{{{\varvec{\Psi}}}_{2} }}{\tau }} \right)B_{{\text{k + 1}}} + \left( {{{\varvec{\Psi}}}_{2} - \frac{{{{\varvec{\Psi}}}_{3} }}{\tau }} \right)(B_{{\text{k}}} - B_{{\text{k + 1}}} )/\tau \hfill \\ \begin{array}{*{20}c} {{\mathbf{F}}_{km} = \frac{{{{\varvec{\Psi}}}_{2} }}{\tau }B_{{\text{k + 1}}} + \frac{{{{\varvec{\Psi}}}_{3} }}{\tau }(B_{{\text{k}}} - B_{{\text{k + 1}}} )/\tau } & {\;{\mathbf{F}}_{0} = {{\varvec{\Psi}}}_{0} } \\ \end{array} . \hfill \\ \end{array} $$

With:

33 $$ \begin{array}{*{20}l} {{\varvec{\Psi}}}_{0} = e^{{{\mathbf{A}}_{0} \tau }} \hfill \\ {{\varvec{\Psi}}}_{1} = \int_{0}^{\tau } {e^{{{\mathbf{A}}_{0} \delta }} } d\delta = {\mathbf{A}}_{0}^{ - 1} \left( {{{\varvec{\Psi}}}_{0} - {\mathbf{I}}} \right) \hfill \\ {{\varvec{\Psi}}}_{2} = \int_{0}^{\tau } \delta e^{{{\mathbf{A}}_{0} \delta }} d\delta = {\mathbf{A}}_{0}^{ - 1} \left( {\tau {{\varvec{\Psi}}}_{0} - {{\varvec{\Psi}}}_{1} } \right) \hfill \\ {{\varvec{\Psi}}}_{3} = \int_{0}^{\tau } {\delta^{2} } e^{{{\mathbf{A}}_{0} \delta }} d\delta = {\mathbf{A}}_{0}^{ - 1} \left( {\tau^{2} {{\varvec{\Psi}}}_{0} - 2{{\varvec{\Psi}}}_{2} } \right) \hfill \\ {{\varvec{\Psi}}}_{4} = \int_{0}^{\tau } {\delta^{3} } e^{{{\mathbf{A}}_{0} \delta }} d\delta = {\mathbf{A}}_{0}^{ - 1} \left( {\tau^{3} {{\varvec{\Psi}}}_{0} - 3{{\varvec{\Psi}}}_{3} } \right) \hfill \\ {{\varvec{\Psi}}}_{5} = \int_{0}^{\tau } {\delta^{4} } e^{{{\mathbf{A}}_{0} \delta }} d\delta = {\mathbf{A}}_{0}^{ - 1} \left( {\tau^{4} {{\varvec{\Psi}}}_{0} - 4{{\varvec{\Psi}}}_{4} } \right). \hfill \\ \end{array} $$

$\gamma_{k + 1}$ can be represented by the status response of the previous moment, its matrix form is

34 $$ \begin{array}{*{20}l} p_{k + 1} = D_{k} p_{k} \Leftrightarrow \hfill \\ \left\{ {\begin{array}{*{20}c} {\gamma_{k + 1} } \\ {\gamma_{k} } \\ {\gamma_{k - 1} } \\ \vdots \\ \vdots \\ {\gamma_{k + 2 - m} } \\ {\gamma_{k + 1 - m} } \\ \end{array} } \right\}{ = }\left[ {\begin{array}{*{20}c} {C_{11} } & {C_{p1} } & {C_{p2} } & \cdots & 0 & {C_{1m} } & {C_{m} } \\ I & 0 & 0 & \cdots & 0 & 0 & 0 \\ 0 & I & 0 & \cdots & 0 & 0 & 0 \\ 0 & 0 & I & \cdots & 0 & 0 & 0 \\ \vdots & \vdots & \vdots & \ddots & \vdots & \vdots & \vdots \\ 0 & 0 & 0 & \cdots & I & 0 & 0 \\ 0 & 0 & 0 & \cdots & 0 & I & 0 \\ \end{array} } \right]\left\{ {\begin{array}{*{20}c} {\gamma_{k} } \\ {\gamma_{k - 1} } \\ {\gamma_{k - 2} } \\ \vdots \\ \vdots \\ {\gamma_{k + 1 - m} } \\ {\gamma_{k - m} } \\ \end{array} } \right\}. \hfill \\ \end{array} $$

With:

35 $$ \begin{array}{*{20}l} {C_{11} = \left( {I - F_{k1} } \right)^{ - 1} \left( {F_{0} + F_{k} } \right)} \hfill & {C_{p1} = \left( {I - F_{k1} } \right)^{ - 1} F_{kp1} } \hfill \\ {C_{p2} = \left( {I - F_{k1} } \right)^{ - 1} F_{kp2} } \hfill & {C_{1m} = \left( {I - F_{k1} } \right)^{ - 1} F_{k1m} } \hfill \\ {C_{m} = \left( {I - F_{k1} } \right)^{ - 1} F_{km} } \hfill & {} \hfill \\ \end{array} . $$

The Floquet transition matrix corresponding $\Phi$ to the state-space expression of the cutting head can be expressed through the mapping function $D_{k}$.

36 $$ \begin{array}{*{20}l} p_{m} = \Phi p_{{0}} \hfill \\ \Phi = D_{m - 1} D_{m - 2} \cdots D_{1} D_{0} . \hfill \\ \end{array} $$

According to Floquet theory, the stability of the system is determined according to the modulus of the eigenvalues of the Floquet transition matrix:

37 $$ \max (|\lambda ({{\varvec{\Phi}}})|)\left\{ {\begin{array}{*{20}l} { < 1,} \hfill & \quad  {{\text{stable}}} \hfill \\ { = 1,} \hfill & \quad  {\text{stability boundary}} \hfill \\ { > 1,} \hfill & \quad  {{\text{unstable}}} \hfill \\ \end{array} } \right.. $$

### Stability verification and analysis

Set the modal parameters in the x, y and z directions of the cutting system to be the same as $k_{x} = 2.76 \times 10^{6}$, $\zeta_{x} = 0.021$, $\omega_{nx} = 209.0\,{\text{Hz}}$; $k_{y} = 2.76 \times 10^{6}$, $\zeta_{{\text{y}}} = 0.021$, $\omega_{{n{\text{y}}}} = 209.0\;{\text{Hz}}$; $k_{z} = 2.76 \times 10^{6}$, $\zeta_{{\text{z}}} = 0.021$, $\omega_{nz} = 209.0\;{\text{Hz}}$.

All the programs in this paper are executed under the platform of MATLAB R2021a on laptop (AMD Ryzen 7 5800H with Radeon Graphics, 3.20 GHz, 32G). To analyze the convergence rate of the improved hybrid fully discrete method (HFDM), the swing speed of the cutting head is set as Vs = 0.5 m/s, the rotation speed of the cutting head is set as n = 45 r/min, the hardness of the coal rock is set as 3.5, the cutting depth is set as 200 mm, 400 mm and 800 mm, respectively, and the cutting width is set as 100 mm, 352 mm and 704 mm, respectively.

**Figure 7:**
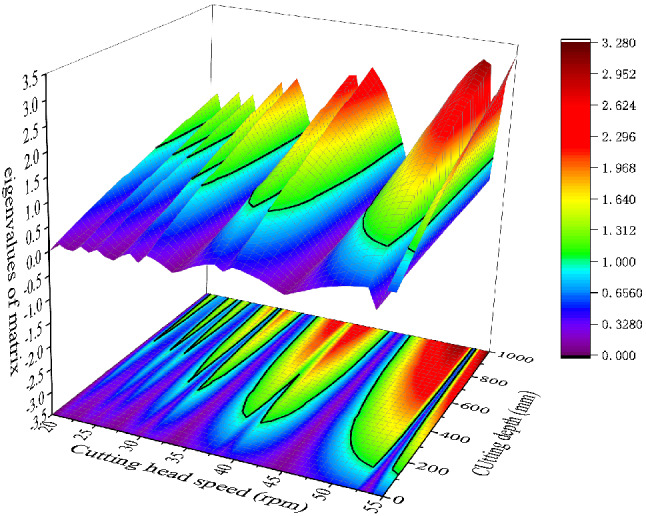
Eigenvalue calculation results of state transition matrix.

When the cutting width is set as 352 mm, the eigenvalue of the transfer matrix calculated by Eq. (36) is shown in Fig. 7. In the figure, the black contour line is used as the boundary line for cutting the stable region. When the combination of cutting depth and cutting speed is below the line, eigenvalue of state transition matrix is less than 1. It indicates that the cutting chatter is stable. When the combination of cutting depth and cutting speed is above the line, it indicates that the calculated cutting chatter is in an unstable state. Therefore, we can reasonably select the cutting depth and cutting speed according to the stability curve to improve the cutting stability and reduce the wear of the pick.

In order to provide a comparison of the accuracy and computational efficiency of different methods. The classical fully discrete method (FDM) and the semi-discrete method (SDM) were considered for further comparison and verification.

To verify the convergence rate of different methods, using the local discrete error method proposed in Refs.^30,31^. Suppose the maximal module of the eigenvalues of the transition matrix $\Phi$ is set as $\left| \mu \right|$, the $\left| {\mu_{{0}} } \right|$ obtained at FDM(m = 200) is set as the exact value. We use the difference between $\left| \mu \right|$ and $\left| {\mu_{{0}} } \right|$ to express the convergence effect $\left| {\left| \mu \right| - \left| {\mu_{{0}} } \right|} \right|$. The convergence speed of the improved mixed discrete method is compared at the beginning. The comparisons of convergence rates among FDM, SDM and HFDM are shown in Fig. 7.

**Figure 8:**
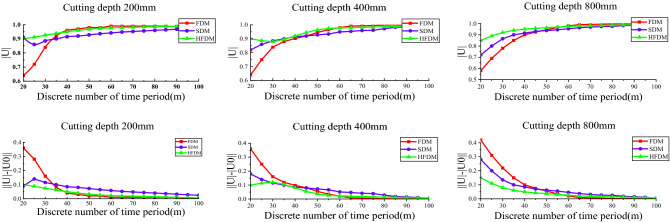
Comparison of convergence rates between different prediction methods.

Figure 8 shows that no matter what the cutting depth is, the discrete local error of the HFDM is significantly smaller than that of others, and with the increase of discrete time parameters m, the convergence rate of HFDM is higher than that of FDM and SDM.

In order to further analyze the prediction accuracy of the proposed HFDM with that of FDM and SDM, as well as the effect of discrete number m on the prediction accuracy, two technical criterias were used. The first is the RMSE mentioned above, and the other is the mean absolute error(MAE)^19^, which can be expressed as:

38 $$ MAE = \frac{1}{n}\sum\limits_{i = 1}^{n} {\left| {y_{i} - \hat{y}_{i} } \right|} . $$

When the FDM (m = 200) is used as a benchmark method to evaluate other numerical methods to predict cutting stability in time domain.

Under different cutting depths, the RMSE and MAE between the eigenvalues $\left| \mu \right|$ and the benchmark $\left| {\mu_{{0}} } \right|$ obtained by FDM, SDM, HFDM methods are shown in the Tables 2 and 3.

**Table 2:** RMSE between different methods.

Cutting depth (mm)	Different methods		
	FDM	HFDM	SDM
200	0.0871	0.0243	0.0218
400	0.0873	0.0218	0.0437
800	0.109	0.0364	0.0680
Average	0.0945	0.0275	0.0445

**Table 3:** MAE between different methods.

Cutting depth (mm)	Different methods		
	FDM	HFDM	SDM
200	0.0212	0.0059	0.0053
400	0.0212	0.0053	0.0106
800	0.0247	0.0088	0.0165
Average	0.0224	0.0067	0.0108

It can be seen from the Table 2 that The RMSE of eigenvalues calculated by HFDM method is lower than other methods. This indicates that under different discrete times m, the dispersion of eigenvalues obtained by HFDM method is lower, indicating that the stability of HFDM calculation is stronger.

In Table 3, the overall MAE of HFDM is also basically lower than that of other methods. It shows that the eigenvalues calculated by HFDM method are closer to the benchmark, that is, the convergence speed of the calculation obtained by using the HFDM method proposed in this paper is faster.

**Figure 9:**
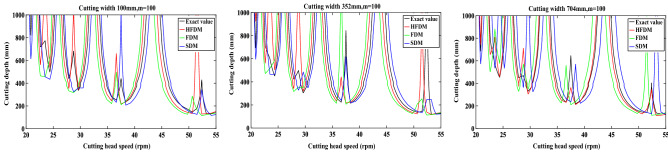
Comparison of the prediction accuracy between different prediction methods.

**Figure 10:**
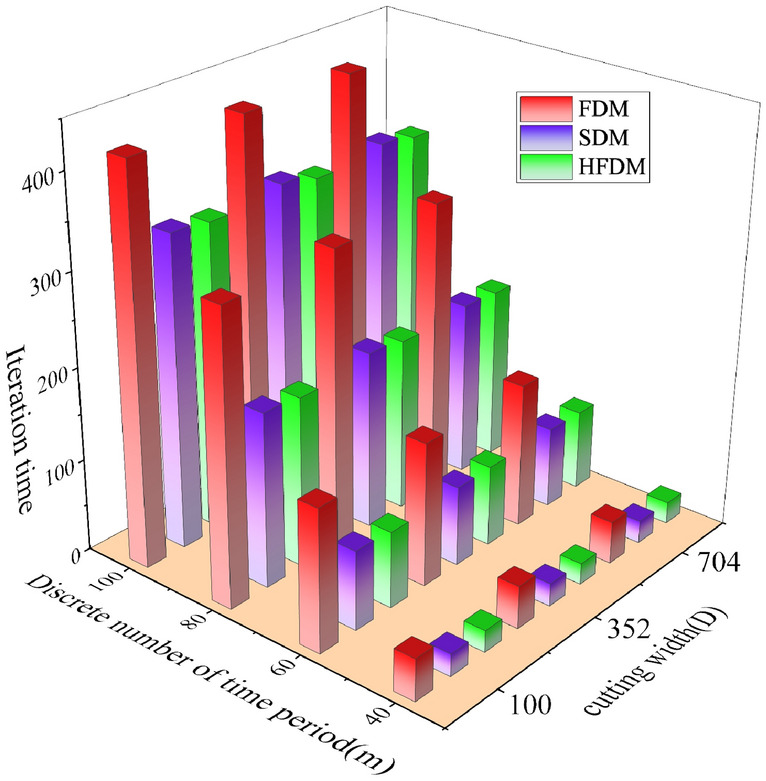
Comparison of calculation time of different prediction methods.

To compare the computational efficiency and computational accuracy of different discrete methods, stability diagrams were determined using three methods for different cutting widths and different discrete time parameters *m*. Other parameters are the same. The cutting width is set as 100 mm, 352 mm, 704 mm respectively. Comparisons of computational efficiency and computational accuracy among FDM, SDM, and HFDM are shown in Figs. 9 and 10.

It is obvious from Fig. 9 that with the change of cutting width, the prediction results of each discrete method have a certain deviation, the average value of RMSE and MAE of each method under different cutting widths were obtained, as shown in Table 4.

**Table 4:** Prediction accuracy between different methods.

Metric	Different methods		
	FDM	HFDM	SDM
RMSE	148.45	135.21	147.52
MAE	99.06	87.85	108.83

It can be seen from Table 4 that the RMSE and MAE of the stability curve calculated by HFDM method are lower than those other methods. It indicates that under the same calculation conditions. The curve error obtained by HFDM method is smaller and the calculation accuracy is higher.

As can be seen from Fig. 10, with the increase of the degree of dispersion m, the calculation time of the cutting stable curve increases gradually, while the cutting depth has little influence on the calculation time. At the same time, comparing the calculation time of different methods, it is found that the calculation time of FDM is about 30% higher than that of HFDM and SDM method, while the calculation time of HFDM and SDM method is not much different under the same parameters.

It can be seen from the above results that the HFDM proposed in this paper is superior to FDM and SDM in convergence speed and calculation accuracy, and is no inferior to SDM in calculation time. The analysis results show that, compared to other discrete methods, the improved discrete method can improve the flutter analysis and prediction efficiency of the cutting head-coal system with almost no loss of calculation accuracy.

## Influencing factors of cutting flutter

The dynamic model of the cutting head-coal system established by the above analysis shows that the dynamic parameters of the cutting system have a certain influence on the prediction of system stability. Excess stiffness and large damping of the cutting system will reduce the vibration amplitude of the cutting head, the loading on the surface of coal and rock will slow down the flutter of the system, improve the system stability, and affect the quality of section formation.

However, the flutter influencing factors of the cutting head were not documented in detail. Thus, the effects of different parameter combinations on the stability of the cutting-coal system are examined by changing the modal stiffness, natural frequency, and damping ratio of the cutting system. The results are presented as follows.

### The effect of modal stiffness

**Figure 11:**
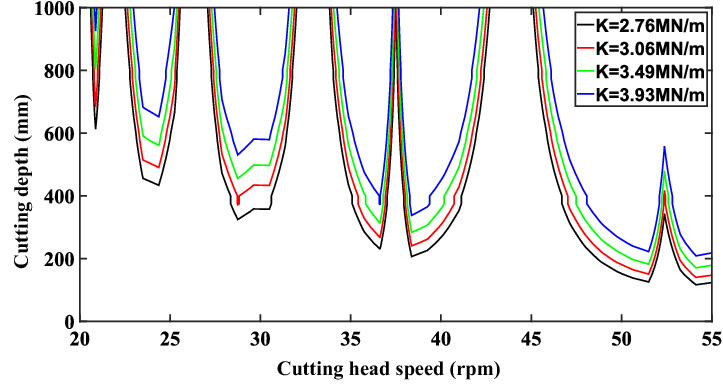
Stability curve of cutting system with different modal stiffness.

According to the system modal stiffness calculation formula: $k = m_{x} \varpi_{n}^{2}$, the modal stiffness of the system is adjusted by changing the modal mass to analyze the influence of the modal stiffness of the system on the stability of the system, and the calculated stability diagram is shown in Fig. 11. It can be seen from the figure that the trend of the diagram between the cutting depth and the cutting speed gradually moves upward with the increase of stiffness, the area of the cutting stability region continues to expand with the increase of stiffness, and the stability of the system is gradually enhanced.

### The effect of system damping ratio

**Figure 12:**
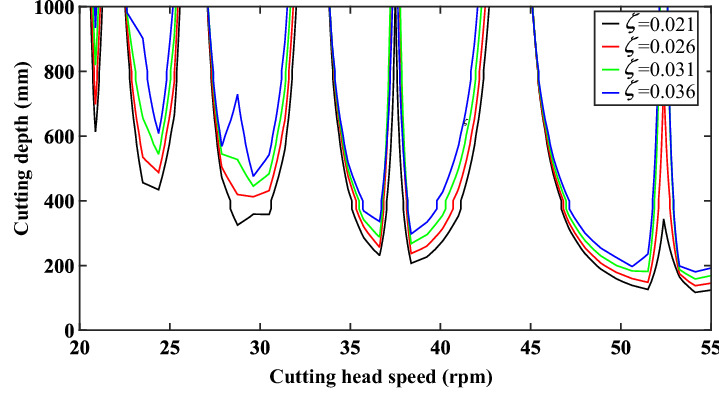
Stability curve of the cutting system with different damping ratios.

To study the relationship between damping ratio and cutting stability, multiple damping ratios were selected, and the calculated stability diagram is shown in Fig. 12. It can be seen from the figure that the influence trend of the modal stiffness of the system is approximately the same. As the damping ratio increases, the cutting stability diagram moves upward and the stability region of the system gradually expands. This means that the system can be improved with the increase of the damping ratio. The flutter stability of the system has been improved.

### The effect of natural system frequency

**Figure 13:**
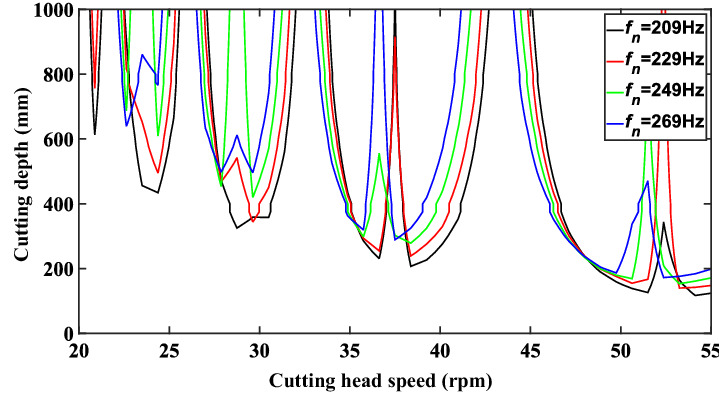
Stability curve of cutting system with different natural frequencies.

In order to analyze the influence of natural frequency on cutting stability, the selected natural frequency values are 209 Hz, 229 Hz, 249 Hz, and 269 Hz. The calculated stability lobe diagram is shown in Fig. 13. It can be seen from the figure that when the natural frequency increases, the stability diagram moves to the left. At the same time, due to the influence of the natural frequency on the stiffness and damping of the system, increasing the natural frequency will also cause the stability diagram to move up gradually, increasing the limit cutting depth at the same speed.

## Experimental study

### Experimental verification of cutting chatter

**Figure 14:**
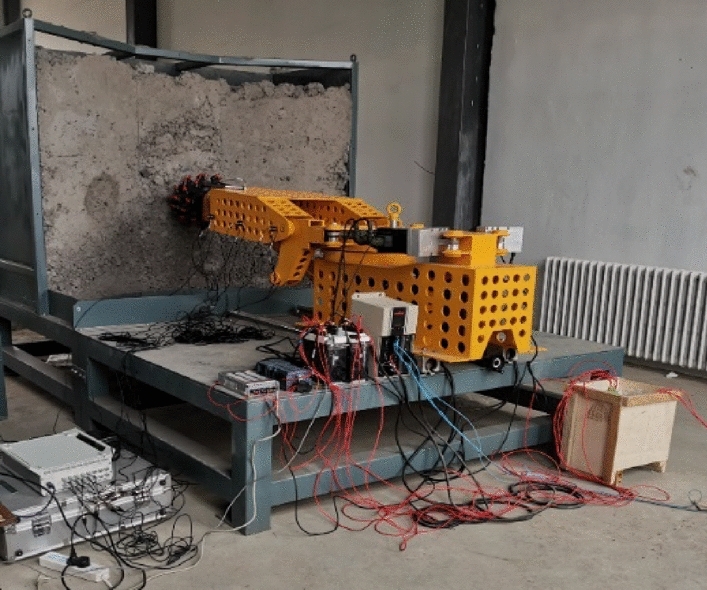
Test bed for the cutting head-coal rock system.

**Figure 15:**
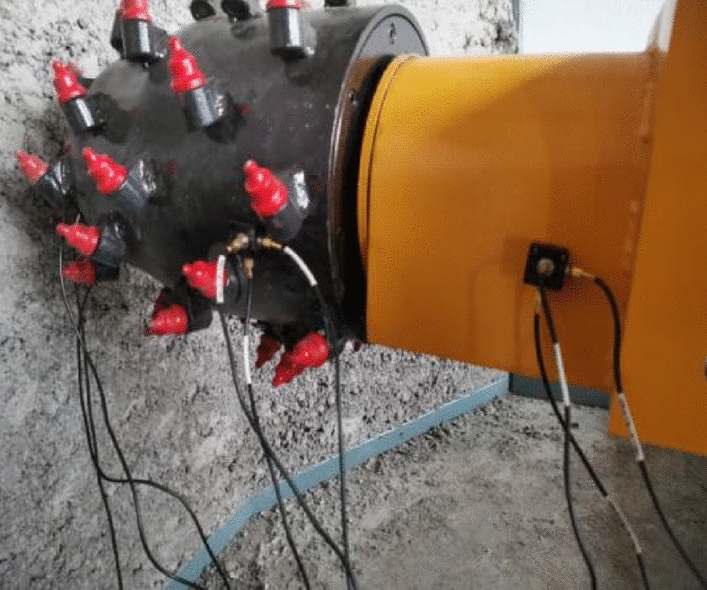
Cutting system measuring point layout.

To verify the correctness of the improved hybrid discrete prediction method, the experimental prototype of the roadheader cutting head and the coal-rock cutting are established on the basis of the similarity criterion. The servo-electric cylinder and variable frequency motor are used by PLC to complete the swing cutting of the cutting head and realize the simulation of actual cutting conditions. DHDAS dynamic test analysis system, developed by Jiangsu Donghua Testing Technology Co., Ltd. The DH311E piezoelectric three-way acceleration sensor, data acquisition instrument, and notebook computer is selected to monitor the actual cutting chatter of the roadheader cutting head experimental prototype, as shown in Fig. 14. Several vibration measuring points are arranged at the cutting head of the experimental cutting head prototype of the roadheader, as shown in Fig. 15.

**Figure 16:**
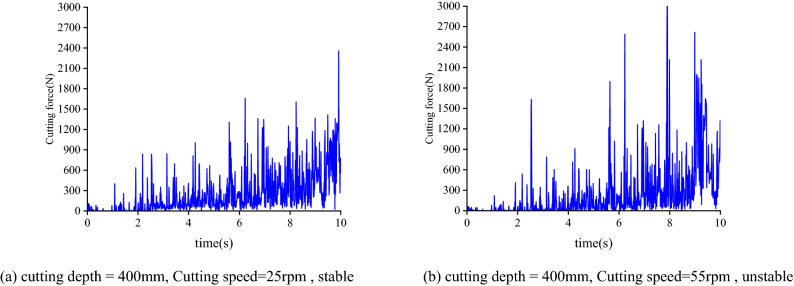
Examples of the measured signals.

In order to verify the correctness of the flutter stability region curve, the cutting force tests were carried out under two working conditions: stable cutting and flutter cutting. The curve of cutting force changing with time is obtained through experiments. The experimental results are shown in Fig. 16. It can be seen from the Fig. 16 that under the condition of chatter cutting, the cutting force curve fluctuates greatly, while the cutting force changes evenly with time during stable cutting.

**Figure 17:**
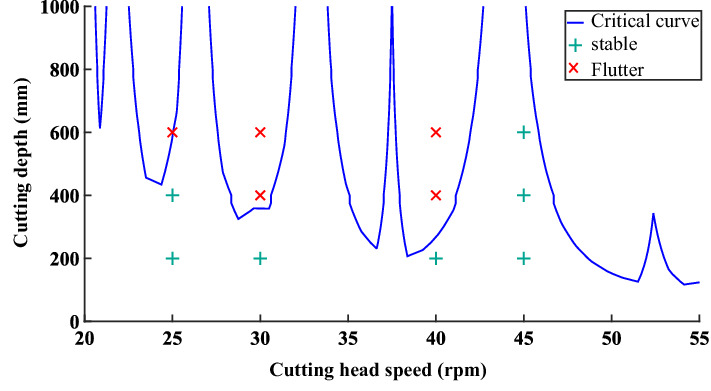
Experimental verification of cutting stability curve.

The flutter stability region curve is established based on the modal coefficients and natural frequencies of the cutting head obtained from the simulation experiment. To verify the correctness of the curve of the flutter stability region, a series of combinations of cutting depth and spindle speed are selected for experimental verification of cutting force under two working conditions of stable cutting and chatter cutting, the results are shown in Fig. 17.

It can be seen from the figure that the experimental results conform to the stability prediction state, indicating that the discrete mixed model established can be well adapted to the cutting stability prediction.

### Cutting wear verification

**Figure 18:**
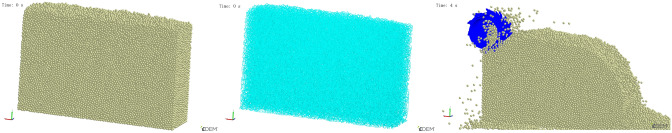
Discrete coal wall cutting model.

Due to the limitations of the test bench device, in order to further explain and verify the influence of cutting chatter stability on cutting head pick wear, Archard Wear model in Edem discrete element simulation was selected for cutting wear verification^32^. Coal wall model was established according to Liu et al.^33^. The bonding model is also used to bond the particles, and the cutting parameters of the cutting head are set to achieve coal wall cutting. The model is established as shown in Fig. 18.

The combination of different cutting depth and cutting speed is selected for cutting simulation. The setting of cutting parameters is shown in Table 5.

**Table 5:** Cutting parameter setting.

	Cutting depth (mm)	Cutting speed (rpm)	Flutter stability
1	400	55	Unstable
2	400	25	Stable
3	800	55	Unstable
4	800	25	Unstable

**Figure 19:**
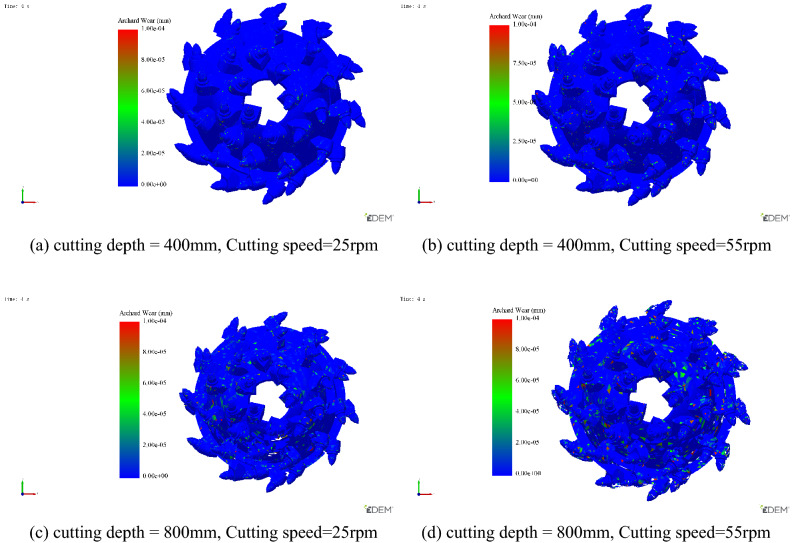
Nephogram of wear depth of cutting head under different cutting parameter.

The above cutting parameters are selected for simulation, and the result Nephogram of cutting head wear depth is shown in Fig. 19.

It can be seen from Fig. 19 above that when the cutting head parameters are in the chatter area, the wear depth of the cutting head is much higher than that of the pick in the stable cutting state. At the same time, the wear of the cutting head increases significantly with the increase of the cutting depth and cutting speed. Export the data of average wear depth of picks with different cutting parameters obtained by simulation, as shown in Table 6.

**Table 6:** Average wear depth of pick with different cutting parameters.

Cutting depth (mm)	Cutting speed (rpm)	Flutter stability	Average wear depth of pick (mm)
400	55	Unstable	0.00198
400	25	Stable	0.00108
800	55	Unstable	0.01468
800	25	Unstable	0.00727

It can be seen from the above table that, keeping the cutting depth unchanged, under the condition that the cutting head speed is nearly doubled, the cutting head wear is nearly doubled, while keeping the cutting speed unchanged, under the condition that the cutting head depth is doubled, the cutting head wear is increased by 600%, this indicates that the influence of cutting depth on the cutting tooth wear is much higher than the cutting head speed.

It can be seen from the above simulation analysis, According to the cutting stability curve, the stable cutting parameter combination below the curve can effectively reduce the cutting wear of the cutting head. This proves the validity and feasibility of the established model, which can provide a basis for extending the service life of the equipment.

## Conclusions and future work

This paper focuses on the prediction of cutting stability in the coal and rock cutting process using the HFDM method, and the following conclusions can be drawn from this research.

1. An improved discrete method based on the mixed Newton Lagrange interpolation is selected to obtain the solution of the inhomogeneous term of the dynamic flutter force in the cutting dynamic equation. The stability diagram representing the critical stability of cutting flutter is constructed and compared with conventional discrete methods for verification based on floquet theory. The results show that the convergence rate, prediction accuracy, and prediction efficiency of the HFDM discrete method are higher than those of the full discrete method and the semi-discrete method.
2. The influence of the system modal mass, stiffness, and damping ratio on the stability of the cutting system is studied based on the HFDM discrete method. The results show that when other system parameters remain constant, improving the modal stiffness, damping ratio, and natural frequency of the system can effectively improve the flutter stability of the cutting system.
3. An experimental prototype of cutting coal rock is established to carry out the cutting stability test of the cutting head coal rock system. The results show that the dynamic model of multiple interaction effect and velocity effect between the cutting head and the coal rock and the stability diagram obtained by the improved hybrid discrete method can be very close to the actual cutting state, which verifies the effectiveness of the improved hybrid discrete method and dynamic model.
4. The method of Edem discrete element simulation is used to simulate the cutter wear. The results show that selecting the cutting parameters in the flutter stability area can effectively reduce the cutter wear and prolong the service life of the equipment, which provides a basis for the reasonable selection of the cutting parameters of the roadheader.

It should be noticed that the research on cutting wear is currently obtained through numerical simulation, In the future research process. It is necessary to study the influence of cutting chatter on the wear of cutting head and cutting surface roughness through experiments. The GPU parallel computing method can also be considered to improve the calculation efficiency.

## Data Availability

The datasets used and/or analysed during the current study available from the corresponding author on reasonable request.
